# Supplementary material for: The causal role of circulating inflammatory markers in osteoporosis: a bidirectional Mendelian randomized study
Source: Front Immunol. 2024 Jul 18;15:1412298. doi: 10.3389/fimmu.2024.1412298 (PMC11291241; doi:10.3389/fimmu.2024.1412298)
Supplement: Supplementary file 1 [file DataSheet_1.zip › supplementary material/OSTEOPOROSIS-CIM/GCST90274761.tsv.gz_forest.pdf]

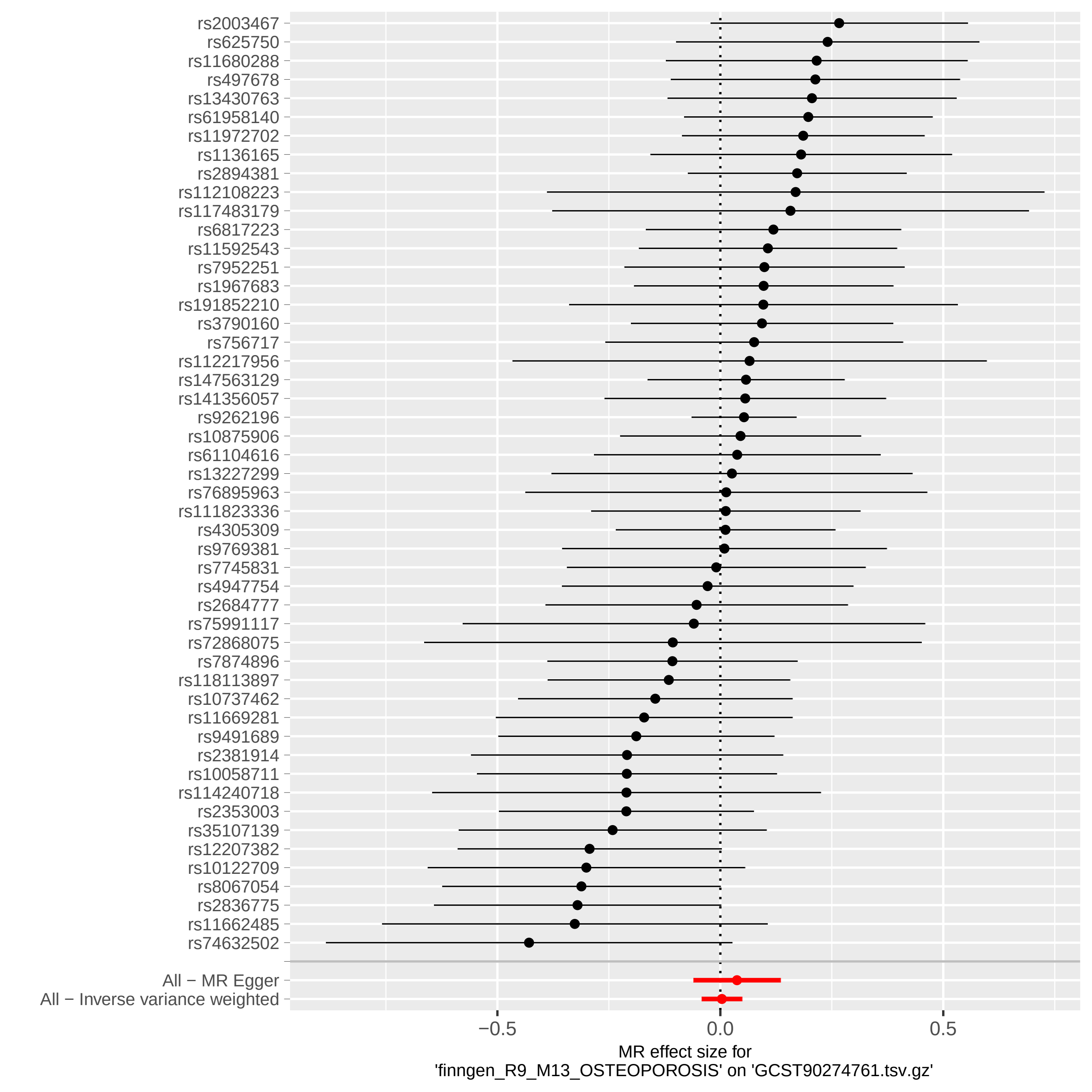
rs2003467
rs625750
rs11680288
rs497678
rs13430763
rs61958140
rs11972702
rs1136165
rs2894381
rs112108223
rs117483179
rs6817223
rs11592543
rs7952251
rs1967683
rs191852210
rs3790160
rs756717
rs112217956
rs147563129
rs141356057
rs9262196
rs10875906
rs61104616
rs13227299
rs76895963
rs111823336
rs4305309
rs9769381
rs7745831
rs4947754
rs2684777
rs75991117
rs72868075
rs7874896
rs118113897
rs10737462
rs11669281
rs9491689
rs2381914
rs10058711
rs114240718
rs2353003
rs35107139
rs12207382
rs10122709
rs8067054
rs2836775
rs11662485
rs74632502
All − MR Egger
All − Inverse variance weighted
−0.5
0.0
0.5
MR effect size for
'finngen_R9_M13_OSTEOPOROSIS' on 'GCST90274761.tsv.gz'
